# FKBP12.6 protects heart from AngII‐induced hypertrophy through inhibiting Ca^2+^/calmodulin‐mediated signalling pathways in vivo and in vitro

**DOI:** 10.1111/jcmm.13645

**Published:** 2018-04-22

**Authors:** Yun‐Fei Xiao, Zhi‐Xiong Zeng, Xiao‐Hui Guan, Ling‐Fang Wang, Chan‐Juan Wang, Huidong Shi, Weinian Shou, Ke‐Yu Deng, Hong‐Bo Xin

**Affiliations:** ^1^ Institute of Translational Medicine Nanchang University Nanchang China; ^2^ School of Life Science Nanchang University Nanchang China; ^3^ Georgia Cancer Center Augusta University Augusta GA USA; ^4^ Riley Heart Research Center Herman B Wells Center for Pediatric Research Indiana University School of Medicine Indianapolis IN USA

**Keywords:** angiotensin II, Ca^2+^ signalling, cardiac hypertrophy, FKBP12.6, transgenic mice

## Abstract

We previously observed that disruption of FK506‐binding protein 12.6 (FKBP12.6) gene resulted in cardiac hypertrophy in male mice. Studies showed that overexpression of FKBP12.6 attenuated thoracic aortic constriction (TAC)‐induced cardiac hypertrophy in mice, whereas the adenovirus‐mediated overexpression of FKBP12.6 induced hypertrophy and apoptosis in cultured neonatal cardiomyocytes, indicating that the role of FKBP12.6 in cardiac hypertrophy is still controversial. In this study, we aimed to investigate the roles and mechanisms of FKBP12.6 in angiotensin II (AngII)‐induced cardiac hypertrophy using various transgenic mouse models in vivo and in vitro. FKBP12.6 knockout (FKBP12.6^−/−^) mice and cardiac‐specific FKBP12.6 overexpressing (FKBP12.6 TG) mice were infused with AngII (1500 ng/kg/min) for 14 days subcutaneously by implantation of an osmotic mini‐pump. The results showed that FKBP12.6 deficiency aggravated AngII‐induced cardiac hypertrophy, while cardiac‐specific overexpression of FKBP12.6 prevented hearts from the hypertrophic response to AngII stimulation in mice. Consistent with the results in vivo, overexpression of FKBP12.6 in H9c2 cells significantly repressed the AngII‐induced cardiomyocyte hypertrophy, seen as reductions in the cell sizes and the expressions of hypertrophic genes. Furthermore, we demonstrated that the protection of FKBP12.6 on AngII‐induced cardiac hypertrophy was involved in reducing the concentration of intracellular Ca^2+^ ([Ca^2+^]i), in which the protein significantly inhibited the key Ca^2+^/calmodulin‐dependent signalling pathways such as calcineurin/cardiac form of nuclear factor of activated T cells 4 (NFATc4), calmodulin kinaseII (CaMKII)/MEF‐2, AKT/Glycogen synthase kinase 3β (GSK3β)/NFATc4 and AKT/mTOR signalling pathways. Our study demonstrated that FKBP12.6 protects heart from AngII‐induced cardiac hypertrophy through inhibiting Ca^2+^/calmodulin‐mediated signalling pathways.

## 1 INTRODUCTION

Cardiac hypertrophy is characterized with myocardial cell hypertrophy, extracellular matrix increase and myocardial interstitial cell proliferation, and it is a compensatory response to variety of cardiovascular diseases such as high blood pressure, heart valve disease and myocarditis. Many factors including mechanical stress and neurohumoural stimulation induce cardiac hypertrophy.^1^ Among them, angiotensin II (AngII), a degraded peptide from the renin‐angiotensin system, is one of the most powerful stimuli in inducing cardiac hypertrophy and fibrosis.^2^ In patients with an excessive activated RAS (rennin‐angiotensin system) or enhanced responsiveness to AngII, the inappropriate remodelling and vascular diseases were initiated.^3^

The roles of AngII in cardiovascular system are mainly mediated by AT1R activation via complex interacting signalling pathways involving primary stimulation of phospholipase C (PLC), Ca^2+^ mobilization, secondary activation of non‐receptor tyrosine kinase Src, phosphatidylinositol 3‐kinase (PI3‐kinase)/protein kinase B (AKT) and mitogen‐activated protein kinase.^3^, ^4^, ^5^ It has been reported that AngII‐activated Ca^2+^ signalling pathway initiated the progress of cardiomyocyte hypertrophy.^4^, ^6^ AngII induces Ca^2+^ influx *via* G protein‐mediated activation of PLC that leads to phosphatidylinositol hydrolysis and formation of inositol trisphosphate (IP3) and diacylglycerol (DAG), in which IP3 binds to its receptor on sarcoplasmic reticulum (SR), opening the channel that allows calcium efflux into the cytoplasm.^3^, ^5^

FK506‐binding protein 12.6 (FKBP12.6), a member of the FKBPs family, has 85% amino acid sequence identity with FKBP12^7^ and is selectively associated with cardiac ryanodine receptor (RyR2) which is predominantly expressed in heart and plays a crucial role in the regulation of Ca^2+^ release from cardiac SR.^8^, ^9^, ^10^ It has been reported that cyclic ADP Ribose (cADPR) induces a sustained Ca^2+^ increase by dissociating FKBP12.6 from RyR2 of the cardiac SR *via* calcium‐induced calcium release (CICR),^4^ which further activate Ca^2+^‐dependent signalling pathways such as the calcineurin/cardiac form of nuclear factor of activated T cells 4 (NFATc4) and the calmodulin kinaseII (CaMKII)/MEF‐2 pathways to lead to cardiac hypertrophy.^3^, ^5^, ^11^ NFATc4 transcriptional factor, a key molecule in cardiac hypertrophy induction, serves as a down target of calcineurin and Glycogen synthase kinase 3β (GSK3β), in which its transcriptional activity was regulated by its nucleus‐cytoplasmic shuttling.^12^

We have previously showed that disruption of the *FKBP12.6* gene resulted in cardiac hypertrophy in male mice,^13^ and cardiac‐specific overexpression of FKBP12.6 that was driven by the α‐MHC promoter was able to rescue the cardiac hypertrophy and abnormal calcium release in FKBP12.6 knockout (FKBP12.6^−/−^) mice.^14^ Studies showed that cardiac‐specific overexpressing FKBP12.6 in mice blunted thoracic aortic constriction (TAC)‐induced maladaptive left ventricular remodelling and protected against catecholamine‐induced ventricular tachycardia after TAC.^15^, ^16^ The cardiac FKBP12.6 overexpression also reduced the molecular signature of left ventricular hypertrophy (LVH) and the mortality in TAC male mice.^17^ These results suggest that FKBP12.6 may protect heart from cardiac hypertrophy. However, a study showed that the adenovirus‐mediated overexpression of FKBP12.6 induced hypertrophy and apoptosis in cultured neonatal cardiomyocytes,^18^ indicating that the role of FKBP12.6 in cardiac hypertrophy is still controversial.

Several lines of evidence suggest that the renin‐angiotensin system and AngII may contribute to cardiac hypertrophy in response to pressure overload.^19^, ^20^ However, it has been observed that there were disparate effects of ACE inhibitors or disruption of AT1 receptor on the development of LV hypertrophy after pressure overload. These results indicated that it is not clear whether AngII plays a critical role in the development of cardiac hypertrophy induced by pressure overload.^21^, ^22^, ^23^, ^24^, ^25^ Therefore, it is still necessary to clarify the role of FKBP12.6 in AngII‐induced cardiac hypertrophy.

This study aimed to clarify whether FKBP12.6 protects hearts from the AngII‐induced cardiac hypertrophy and to elucidate the mechanisms of FKBP12.6 in pathological cardiac hypertrophy. FKBP12.6^−/−^ mice and the cardiac‐specific FKBP12.6 overexpressing mice were used for producing the mouse models of the pathological cardiac hypertrophy with AngII stimulation. In addition, an H9c2 cell line with stable expressing FKBP12.6 exogenously was constructed for elucidating the mechanisms of the protection of FKBP12.6 in AngII‐induced cellular hypertrophy.

## 2 MATERIALS AND METHODS

### 2.1 Animal models and echocardiography

Two‐month‐old male mice including FKBP12.6^−/−^ mice,^13^ cardiac‐specific FKBP12.6 overexpressing transgenic (FKBP12.6 TG) mice 14 and age‐/genetic‐matched wild‐type (WT) mice were used in this study. The mice with the congenetic background of C57/B6 were prepared by backcrossing both FKBP12.6^−/−^ and FKBP12.6 TG mice with WT mice of C57/B6 genetic background for more than 6 generations, and the WT littermates were used as controls. The mice were anaesthetized with 2.5% 2,2,2‐tribromoethanol (Avertin, Sigma Chemical Co) by intraperitoneal injection (15 μL/g), and each mouse was infused with AngII (1500 ng/kg/min) or saline for 14 days subcutaneously by implantation of an osmotic mini‐pump (ALZET Model 1002D; DURECT, Cupertino, CA, USA) through a small pocket made in the skin between the scapulae.

The echocardiography was measured with the Vevo2100 High‐Resolution Imaging System (Vevo2100; Visual Sonics, Toronto, ON, Canada) for each mouse after anaesthetized with 2% isoflurane and maintained with 1.5% isoflurane. All measurements were covered for at least five consecutive cardiac‐cycles. The values of intraventricular septal thickness (IVS), left ventricular internal dimension (LVID) and left ventricle posterior wall (LVPW) thickness at diastole and systole stage, LV fractional shortening (FS) and LV ejection fraction (EF) were collected and calculated. All animal experiments were approved by the Nanchang University Animal Care Committee and performed according to National Institutes of Health (NIH) guidelines (Guide for the care and use of laboratory animals, 2011).

### 2.2 Histology and immunohistochemistry

Before mice were killed by cervical dislocation, they were exposed to 5% isoflurane until they lost their righting reflex, and the hearts were carefully excised. Then, heart tissues were kept at −80°C immediately or fixed in 4% paraformaldehyde, embedded in paraffin and sectioned at 5‐μm intervals. The cross‐sections were stained with haematoxylin and eosin (H&E) for histological analysis. Picrosirius red staining was used for the histological assessment of collagen types I and III accumulation by polarization microscopy following the instruction of the manufacturer. Wheat germ agglutinin staining was used to evaluate myocyte cross‐sectional area. Briefly, heart sections were de‐paraffinized and incubated with 100 μg/mL FITC‐labelled wheat germ agglutinin (Sigma‐Aldrich, St. Louis, MO, USA) for 30 minutes at 37°C. The cell area was calculated by measuring at least 200 cells per slide.

### 2.3 Preparation of FKBP12.6 overexpression stable H9c2 cell lines

The overexpressing FKBP12.6 stable cell lines were prepared using rat cardiomyocyte H9c2 cells (ATCC^®^ CRL1446^™^, Manassas, VA, USA). Briefly, a full‐length of rat's FKBP12.6 cDNA was amplified with a forward primer (5′‐CGGAATTCATGGGCGTGGAGATCGAGAC‐3′) and a reverse primer (5′‐CCGCTCGAGTCACTCTAAGTTGAGCAGC‐3′) and cloned into the pcDNA3/Flag/1AB eukaryotic expression vector (containing a neomycin‐resistance gene) at the site of EcoR1 and Xho1. Then, H9c2 cells were transfected with pcDNA3/Flag/1AB‐FKBP12.6 vector using SuperFectinTM II In Vitro DNA Transfection Reagent (Shanghai Pufei Biotech Co., Ltd) following the instruction manual. The cells were cultured in a cell incubator (5% CO2, 95% air) at 37°C for 48 hours after transfection and then selected with 800 μg/mL G418 (sigma). After 14‐15 days, single colonies were isolated for expansion and maintained with 400 μg/mL G418 in DMEM supplemented with 10% heat‐inactivated foetal bovine serum. The cells with stable expressing pcDNA3/Flag/1AB‐FKBP12.6 (FKBP12.6 overexpression H9c2 cell line, F12.6) were used for further experiments and the pcDNA3.1/Flag/1AB (Flag‐control H9c2 cell line, Flag) transfected parental cells were used as negative control.

### 2.4 Cell culture and treatment

The overexpressing FKBP12.6 stable H9c2 cells were maintained with 400 μg/mL G418 in DMEM supplemented with 10% heat‐inactivated foetal bovine serum. The cells were passaged every 2‐3 days when the cells were reaching to 70%‐80% confluence. Cultured H9c2 cells of passage 6‐15 were used for our experiments. In different experiments, different cell numbers were used. The cells used for RNA, total protein detection and NFATc4 translocation detection were seeded at 1 × 10^4^ cells/cm^2^ for 24‐hours AngII treatment. The cells used for Phalloidin staining were seeded at 2 × 10^3^ cells/cm^2^ for 24‐hours AngII treatment. The cells used for calcium assay, ROS assay and cell protein phosphorylation were seeded at 2 × 10^4^ cells/cm^2^ for 20‐minutes AngII treatment. The cells were transferred to the DMEM containing 2% FBS for 24 hours at 37°C after overnight culture, and then the cells were stimulated with or without 200 nmol/L AngII (containing 2% FBS) for 20 minutes or 24 hours.

### 2.5 Phalloidin staining

Cells were fixed with 4% polyformaldehyde solution in PBS for 20 minutes at room temperature and then incubated with 0.1% Triton X‐100 in PBS for 5 minutes. The fixed cells were incubated with PBS containing 1% bovine serum albumin (BSA) for 20 minutes. Ten microlitre of Phalloidin‐Tetramethylrhodamine B isothiocyanate (Sigma, P1951) methanolic stock solution were diluted into 200 μL PBS containing 1% BSA as a staining solution and incubated for 30 minutes at room temperature. The cells were washed two or more times with PBS for each step. All the steps were performed away from light. Cell sizes were observed and photographed under fluorescent microscopy (Olympus/BX63). The cell area was calculated by measuring at least 200 cells per slide using Image‐Pro Plus 6 software.

### 2.6 Real‐time quantitative PCR analysis

Total RNA was extracted from hearts or cells with TRIzol (Invitrogen, Carlsbad, CA, USA) and the cDNA was synthesized using M‐MLV reverse transcriptase Kit (Invitrogen). The expressions of target genes were analysed by real‐time quantitative reverse‐transcription polymerase chain reaction (qRT‐PCR) according to the introduction manual (Invitrogen), and normalized by the reference gene GAPDH. The primer pairs (forward, reverse) were used in this study as follows: GAPDH (5′‐AGCCAAAAGGGTCATCATCT‐3′, 5′‐GGGGCCATCCACAGTCTTCT‐3′), FKBP12.6 (5′‐CCAGGAGACGGAAGGACAT‐3′, 5′‐CAAAGATGAGGGTGGCATTG‐3′), ANF (5′‐TCCATCACCAAGGGCTTCT‐3′, 5′‐CCGCTTCATCGGTCTGC‐3′), BNP (5′‐CTCAAAGGACCAAGGCCCTAC‐3′, 5′‐AACCTCAGCCCGTCACAGC‐3′), α‐MHC (5′‐AAGAAGAACTTGGTGCGGCT‐3′, 5′‐ATCGTGCATTTTCTGCTTGGC‐3′), β‐MHC (5′‐GAGACGGACGCCATACAGAG ‐3′, 5′‐ATGCTCTTCCCAGTTGAGCC‐3′).

### 2.7 Western blot analysis

Hearts were dissected and homogenized in RIPA‐buffer (10 μL/mg) containing proteinase inhibitors cocktail (Roche Diagnostics, Mannheim, Germany), the cells were also lysed with RIPA‐buffer. The cytoplasmic and nuclear proteins were isolated using NE‐PER Nuclear and Cytoplasmic Extraction Reagents (Thermofisher Waltham, MA, USA) according to the manufacturer's instructions. The proteins were separated by electrophoresis on 10% to 15% SDS‐PAGE gel and transferred to PVDF membrane. The membrane was blocked with 5% non‐fat milk for 1 hour and incubated at 4°C overnight with the primary antibodies, and then incubated with appropriate secondary antibodies (Thermo fisher, #31460 for rabbit, #31430 for mouse, #A27014 for goat, dilution: 1:5000) at room temperature for 1 hour. Images were obtained by the Bio‐Rad Molecular Imager Chemi Doc XR+ System with Image Lab Software. Expressions of proteins were normalized with the reference GAPDH. The antibodies for Calcineurin (Millipore 07‐068, 1:1000), p‐CaMKII (Thr286/287, millipore 06‐881, 1:1000), total CaMKII (Abcam, ab52476, 1:2000), TBP (Abcam, ab818, 1:1000), NFATc4 (Abcam, ab62613, 1:1000), MCIP1 (sc377507, 1:200), α‐tubulin (sc5286, 1:1000), TGFβ1 (sc‐146, 1:200), BNP (sc‐67455, 1:200) (Santa cruz), Bcl2 (Gene Tex, GTX100064, 1:1000), FKBP12.6 (Thermo pierce antibodies, PA1‐026A, 1:1000), p‐AKT (Ser473, #4060), p‐mTOR (Ser2448, #5536), p‐GSK3β (Ser9, #9336), p‐Smad3 (Ser423/425, #9520), p‐ERK1/2 (Thr202/Tyr204, #9101), total AKT (#9272), mTOR (#2983), GSK3β (#9315), Smad3 (#9523), ERK1/2 (#9102), Bax (#2772, Cell Signaling Technology, 1:1000), GAPDH (BBI, Shanghai, China, D190636, 1:5000) were used in this study.

### 2.8 Calcium assay

After 20‐minutes Ang II (200 nmol/L) treatment, cells were collected and incubated with 5 μmol/L Fluo3‐AM (Sigma) at 37°C for 30 minutes, and then the cells were washed three times with PBS and further incubated for 40 minutes before flow cytometry assay. The concentrations of intracellular Ca^2+^ ([Ca^2+^]i) were determined by measuring the green fluorescent density and normalized with negative control. Five thousand cells were analysed for each sample. All the steps were performed away from light.^26^, ^27^

### 2.9 ROS detection

After 20‐minutes Ang II (200 nmol/L) treatment, the intracellular ROS contents were measured by staining with 2, 7‐dihydrodichlorofluorescein diacetate (H2DCF‐DA, Sigma) and monitoring the fluorescent dichlorofluorescein. Cells were incubated with 10 μmol/L H2DCF‐DA for 20 minutes. Then, the cells were washed twice with PBS and collected for flow cytometry assay. Five thousand cells were analysed for each sample. All the steps were performed away from light.

### 2.10 Apoptosis detection

Apoptosis was determined by Annexin V and propidium iodide (PI) double staining (DOJINDO, AD10). After 20‐minutes AngII (200 nmol/L) treatment, cells were detached with trypsin‐EDTA, washed twice with PBS and resuspended in 500 μL of 1 ×  binding buffer. The cells were then stained with Annexin V‐FITC and PI according to the manufacturer's instructions. Apoptosis rates were determined by flow cytometry. Ten thousand cells were analysed for each sample. All the steps were performed away from light.

### 2.11 Data analysis

Independent two‐sample *t*‐test and two‐way ANOVA were applied to determine statistical significance. Differences were considered to be statistically significant when *P *< .05.

## 3 RESULTS

### 3.1 FKBP12.6 deficiency aggravates AngII‐induced cardiac hypertrophy in vivo

**Figure 1:**
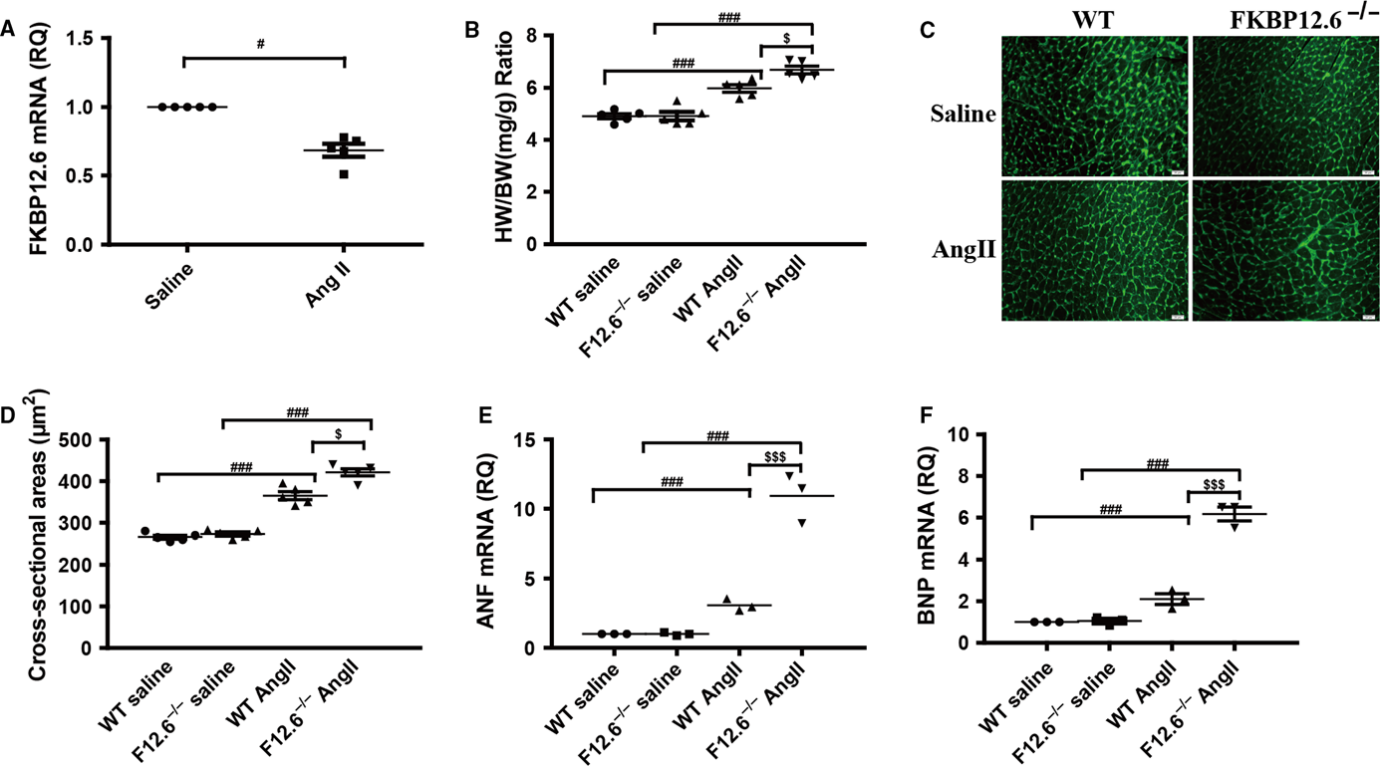
FKBP12.6 deficiency aggravates AngII‐induced cardiac hypertrophy in vivo. FKBP12.6^−/−^ and WT mice were subjected to 14 d AngII or saline infusion, and then the hearts were isolated from the mice for histological and biochemical analyses. The mRNA expressions of FKBP12.6 in hearts from WT mice after AngII or saline infusion were determined by qRT‐PCR (A). The heart weight and body weight of each mouse were measured for calculating the ratio of heart weight/body weight (HW/BW) (B). The myocyte cross‐sectional areas were stained with FITC‐conjugated wheat germ agglutinin (C), and quantitatively analysed (D, ×400, n = 200 cells per section). The mRNA expressions of hypertrophic makers ANF (E) and BNP (F) were analysed by qRT‐PCR. The data represent the mean ± SEM, n = 5 mice, ^$^ *P *< .05, ^$$^ *P *< .01 for FKBP12.6^−/−^ vs WT, and ^#^ *P *< .05, ^##^ *P *< .01, ^###^ *P *< .001 for saline vs AngII infusion groups

To investigate the effects of FKBP12.6 on cardiac hypertrophy, we first examined the expressions of FKPB12.6 in the heart of WT mice in response to AngII stimulation. As showed in Figure 1A, the mRNA levels of FKBP12.6 were down‐regulated by 29% in hearts of WT mice after AngII infusion for 14 days, suggesting that FKBP12.6 might play a critical role in AngII‐induced cardiac hypertrophy. Given that the expression of FKBP12.6 was decreased in the hearts of WT mice after AngII stimulation, the effects of FKBP12.6 deficiency on AngII‐induced cardiac hypertrophy were further investigated using FKBP12.6^−/−^ mice. As showed in Figure 1B, AngII infusion for 14 days significantly increased the HW/BW ratio in FKBP12.6^−/−^ mice by 37.9% compared with the 27.2% augment in WT hearts, due to the heart weights of FKBP12.6^−/−^ mice were significantly increased compared with the WT hearts, while the body weights of both mice were not influenced by AngII stimulation (Table S1), indicating that FKBP12.6 might protect heart from AngII‐induced cardiac hypertrophy. Therefore, we further checked the size of cardiomyocytes and the expressions of hypertrophic genes after AngII stimulation. As showed in Figure 1C,D, the cross‐sectional areas of cardiomyocytes in FKBP12.6^−/−^ mice were significantly augmented compared to WT mice after Ang II infusion. Consistent with the above result, disruption of FKBP12.6 significantly increased AngII‐induced expressions of ANF and BNP which served as the hallmarks of cardiac hypertrophy (Figure 1E,F). In addition, although we observed that AngII stimulation markedly promoted the fibrosis of cardiac tissues in WT mice, there was no significant aggravation of the fibrosis in the null mice (Figure S1). To examine the effects of FKBP12.6 deficiency on cardiac functions after AngII infusion, echocardiography was measured for each mouse. As analysed in Table S1, the IVS thickness and the left ventricle (LV) mass in FKBP12.6^−/−^ mice were significantly augmented compared with WT hearts after AngII infusion (IVS D increased 52.6% vs 32.7%, IVS S increased 37.9% vs 20.8%, LV mass increased 41.0% vs 34.1, respectively, ^$^ *P *< .05, n = 5). Thus, our results indicated that FKBP12.6 deficiency aggravated the AngII‐induced cardiac hypertrophy in vivo.

### 3.2 Cardiac‐specific overexpression of FKBP12.6 protects hearts from AngII‐induced cardiac hypertrophy in vivo

**Figure 2:**
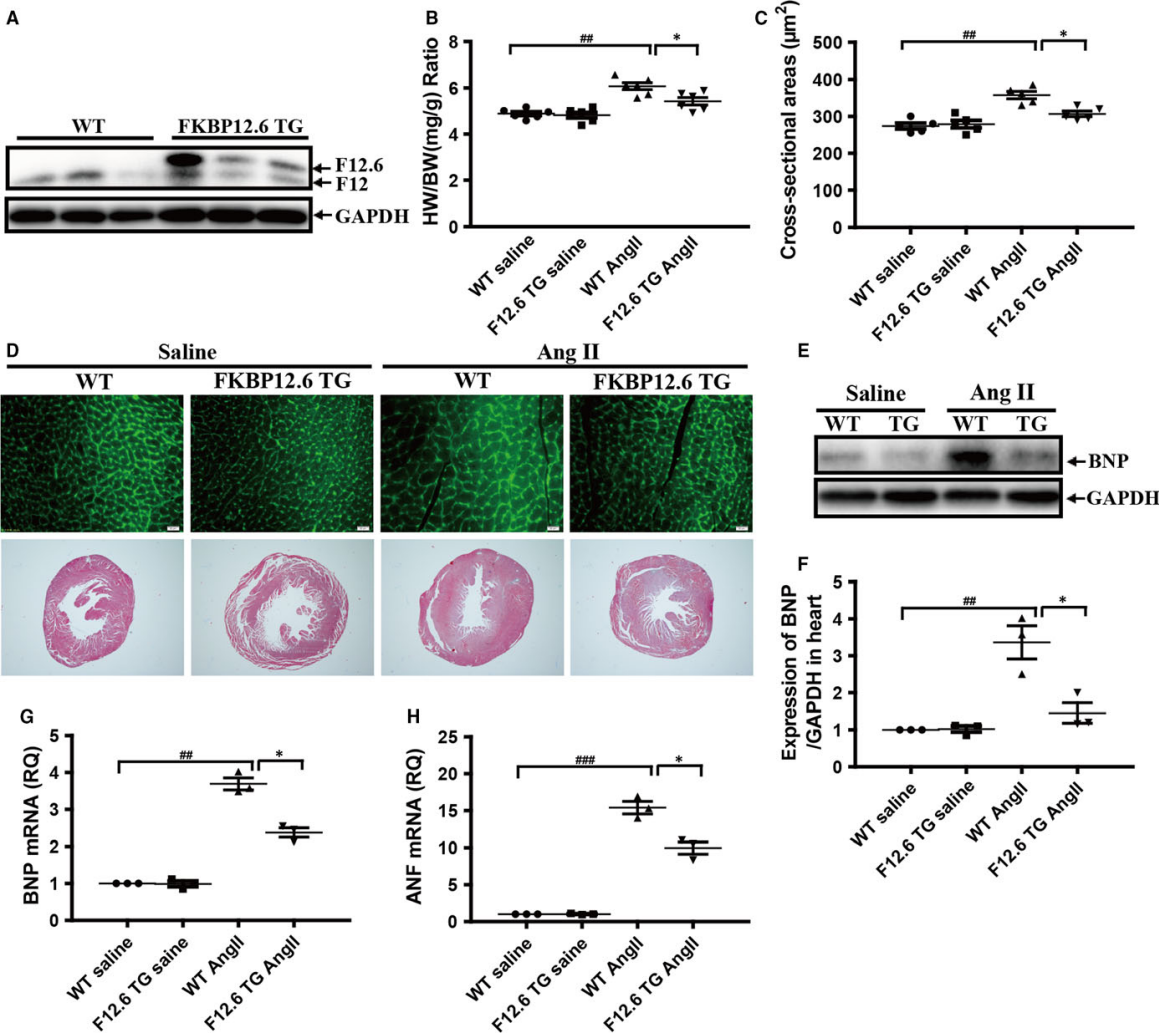
Cardiac‐specific overexpression of FKBP12.6 protects hearts from AngII‐induced cardiac hypertrophy in vivo. The expressions of FKBP12.6 protein in the hearts from three lines 2‐month‐old FKBP12.6 transgenic mice (FKBP12.6 TG) and their WT littermates were determined by Western blot (A). The heart weight and body weight of each mouse were measured for calculating the ratio of heart weight/body weight (HW/BW) in FKBP12.6 TG and WT mice which were subjected to 14 days AngII or saline infusion (B). The myocyte cross‐sectional areas were determined by FITC‐conjugated wheat germ agglutinin staining (D, upper, ×400) and quantitative analysis (C, n = 200 cells per section), and the histological examination was analysed by HE staining (D, lower, ×40). The expressions of BNP protein (E&F) and the mRNA expressions of hypertrophic makers BNP (G) and ANF (H) in hearts from FKBP12.6 TG and WT mice after AngII or saline infusion were analysed by Western blot and qRT‐PCR. The data represent the mean ± SEM, n = 5, *P < .05 for FKBP12.6 TG vs WT mice, and ^##^ *P *< .01, ^###^ *P *< .001 for saline vs AngII infusion groups

Next, the cardiac‐specific overexpression of FKBP12.6 transgenic (FKBP12.6 TG) mice driven by α‐MHC promoter was used for further confirming the effects of FKBP12.6 on AngII‐induced cardiac hypertrophy. The overexpressions of FKBP12.6 in hearts were confirmed by Western blot in three different lines of FKBP12.6 TG mice (Figure 2A). The mouse line that expressed the highest level of FKBP12.6 was used in all of the following studies. The results showed that overexpression of FKBP12.6 in heart significantly inhibited AngII‐induced increase in heart weight, seen as the HW/BW ratios of FKBP12.6 TG mice were significantly decreased compared with that of WT mice (12.4% vs 24.4%, *P < .05, n = 5) after 14 days AngII infusion (Figure 2B). FITC‐conjugated wheat germ agglutinin staining and H&E staining also showed that the FKBP12.6 TG mice markedly attenuated the AngII‐induced the increases of the cardiomyocyte size and ventricular wall thickness (Figure 2C,D). The expressions of the hypertrophic markers such as BNP (Figure 2E‐G) and ANF (Figure 2H) in the hearts of the TG mice were markedly lower than those of WT hearts. However, FKBP12.6 overexpression seems no effect on AngII‐induced fibrosis (Figure S2). Consistent with the above studies, the results of echocardiography (Table S2) also showed that FKBP12.6 TG mice protected heart from AngII‐induced hypertrophy, seen as the decreased heart weight, thickness of IVSD, IVSS and LV mass. All the results indicated that overexpression of FKBP12.6 protected heart from AngII‐induced cardiac hypertrophy in mice.

### 3.3 Cardiac‐specific overexpression of FKBP12.6 markedly inhibited AngII‐induced elevation of calcineurin, CaMKII and AKT activities in mice

**Figure 3:**
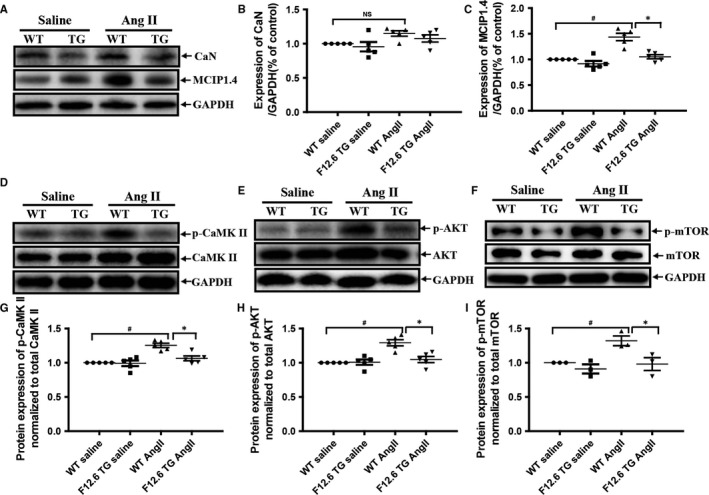
Cardiac‐specific overexpression of FKBP12.6 markedly inhibited AngII‐induced elevation of calcineurin, CaMKII and AKT activities in mice. The expressions of calcineurin (CaN) and MCIP1.4 (a highly sensitive readout for calcineurin activity) were detected by Western blot (A) and quantitatively determined by density analysis (B,C) in hearts from FKBP12.6 TG and WT mice that were subjected to 14 days of AngII or saline infusion. The expressions of the total or phosphorylated proteins including CaMKII, AKT and mTOR were detected by Western blot analysis (D‐F) and the protein expression of p‐CaMKII (G), p‐AKT (H) and p‐mTOR (I) was normalized to total CaMKII, AKT and mTOR in the hearts of the mice. GAPDH expression was used as loading control. The data represent the mean ± SEM, n = 5, *P < .05 for FKBP12.6 TG vs WT mice and ^#^ *P *< .05 for saline vs AngII infusion groups, NS represents no significance

The activities of the Ca^2+^‐dependent signalling pathways such as the calcineurin/NFATc4 and the CaMKII/MEF‐2 pathways are closely associated with the development of cardiac hypertrophy.^11^ In addition, it has been reported that AKT/mTOR pathways were also involved in AngII‐induced cardiac hypertrophy signaling.^3^ So, the activities of calcineurin, CaMKII, AKT and mTOR were checked to elucidate the mechanisms of the protection of FKBP12.6 on AngII‐induced cardiac hypertrophy. Our results showed that there was no significant difference in the expression of calcineurin (CaN) (Figure 3A,B) in hearts of WT and FKBP12.6 TG mice after AngII infusion. However, the expression of MCIP 1.4 which serves as a highly sensitive readout for calcineurin activity was markedly increased in WT but declined in FKBP12.6 TG hearts after AngII stimulation (Figure 3A,C), suggesting that the Ca^2+^/calmodulin/calcineurin‐dependent signalling pathways might be altered. Our results also showed that cardiac‐specific overexpression of FKBP12.6 markedly alleviated the increased expressions of the phosphorylated CaMKII (indicative of CaMKII activation, Figure 3D,G), phosphorylated AKT (indicative of AKT activation, Figures 3E,H) and the phosphorylated mTOR (indicative of mTOR activation, Figure 3F,I) induced by AngII infusion, although the expressions of the total CaMKII, AKT and mTOR were not altered (Figure 3D‐I, P* *< .05, n = 3). All of the results indicated that the overexpressions of FKBP12.6 in hearts would protect heart from AngII‐induced cardiac hypertrophy through inhibiting calcium‐related signalling pathways.

### 3.4 Overexpression of FKBP12.6 protects H9c2 cells from AngII‐induced hypertrophy

**Figure 4:**
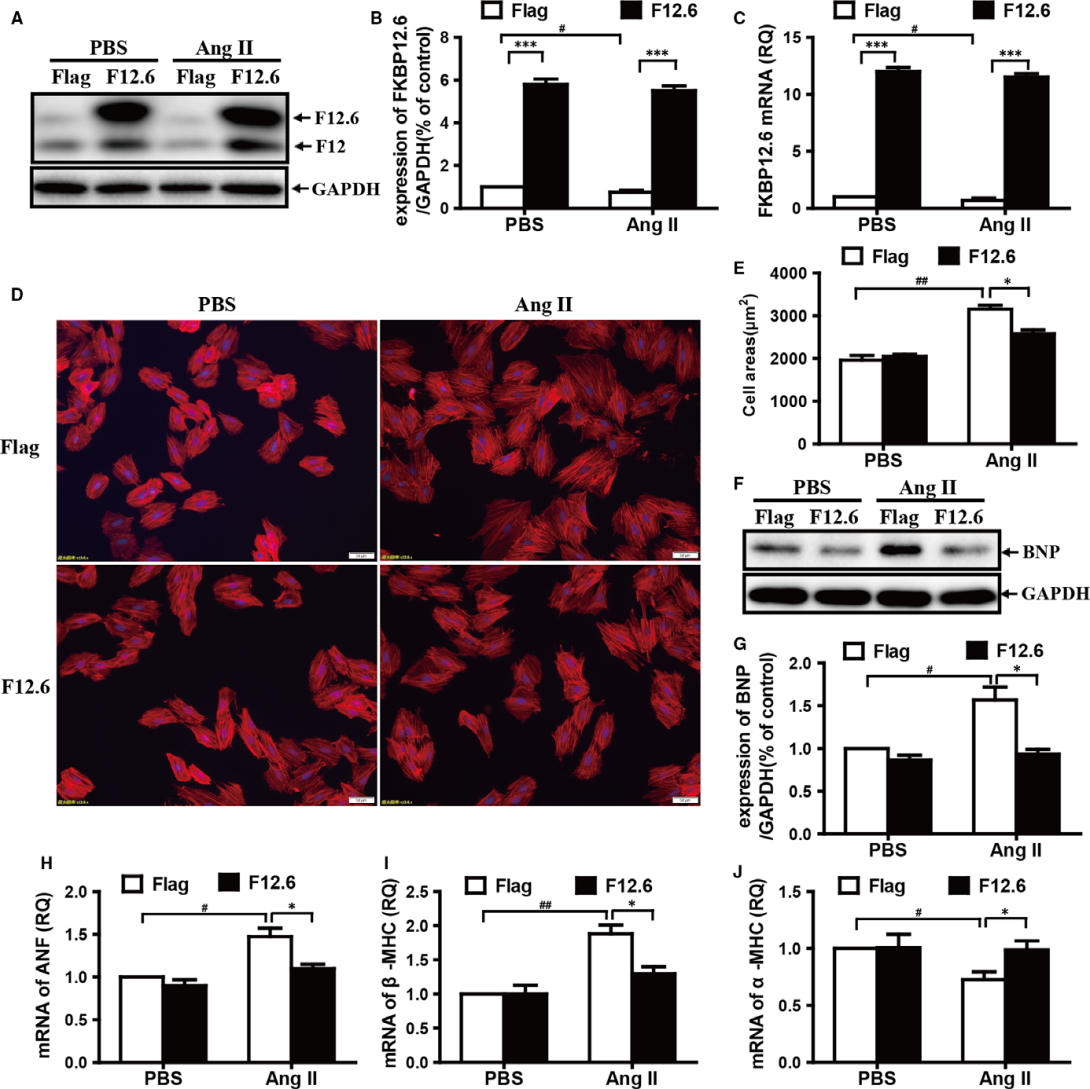
Overexpression of FKBP12.6 protects H9c2 cells from AngII‐induced hypertrophy. FKBP12.6 overexpressing stable H9c2 cell lines were prepared as described in the methods, and both FKBP12.6 overexpressing (F12.6) and Flag‐control (Flag) H9c2 cells were treated with or without 200 nmol/L AngII for 24 h. The expressions of FKBP12.6 proteins were detected by Western blot (A) and quantitatively determined by density analysis (B). The mRNA expression of FKBP12.6 was also examined by qRT‐PCR (C). The cell sizes of FKBP12.6 overexpression (F12.6) and Flag‐control (Flag) H9c2 cells were detected by Phalloidin‐Tetramethylrhodamine staining (D, ×200), and the cell area was calculated by measuring at least 200 cells per slide using Image‐Pro Plus 6 software (E). The expressions of BNP protein (F&G) and the mRNA expression of ANF (H), β‐MHC (I) and α‐MHC (J) were determined by Western blot analysis and qRT‐PCR, respectively. All results were presented from three independent experiments, and the data represent the mean ± SEM, *P < .05, ***P < .001 for FKBP12.6‐overexpression vs Flag‐control groups and ^#^ *P *< .05, ^##^ *P *< .01 for PBS vs AngII groups

To further study the protective roles and mechanisms of FKBP12.6 on AngII‐induced hypertrophy in vitro, the overexpressing FKBP12.6 stable H9c2 cell lines (F12.6) were prepared and the expressions of FKBP12.6 were confirmed by Western blot and qRT‐PCR. The protein and mRNA levels of FKBP12.6 in Flag‐control cells were down‐regulated by 24% and 30%, respectively after AngII administration for 24 hours (Figure 4A‐C). H9c2 cells were stained with Phalloidin‐Tetramethylrhodamine and as shown in Figure 4D,E, the cardiomyocyte sizes have no differences between the Flag‐control (Flag) and overexpression of FKBP12.6 (F12.6) H9c2 cells. And as we expect, Flag‐control H9c2 cells (Flag) treated with AngII were much larger than those of the untreated cells, whereas the overexpression of FKBP12.6 greatly alleviated the AngII‐induced myocyte hypertrophy in H9c2 cells (61.0% vs 25.8%, *P < .05). In addition, we also observed that overexpression of FKBP12.6 was able to prevent the increases of BNP protein expression (Figure 4F,G), the transcription level of ANF (Figure 4H) and β‐MHC (Figure 4I), and the reduced α‐MHC expression after Ang II stimulation (Figure 4J).

### 3.5 Overexpression of FKBP12.6 significantly reduces the elevation of the intracellular Ca^2+^ concentrations ([Ca^2+^]i) and inhibits the activity of Ca^2+^/calcineurin signalling pathways after AngII stimulation in H9c2 cells

**Figure 5:**
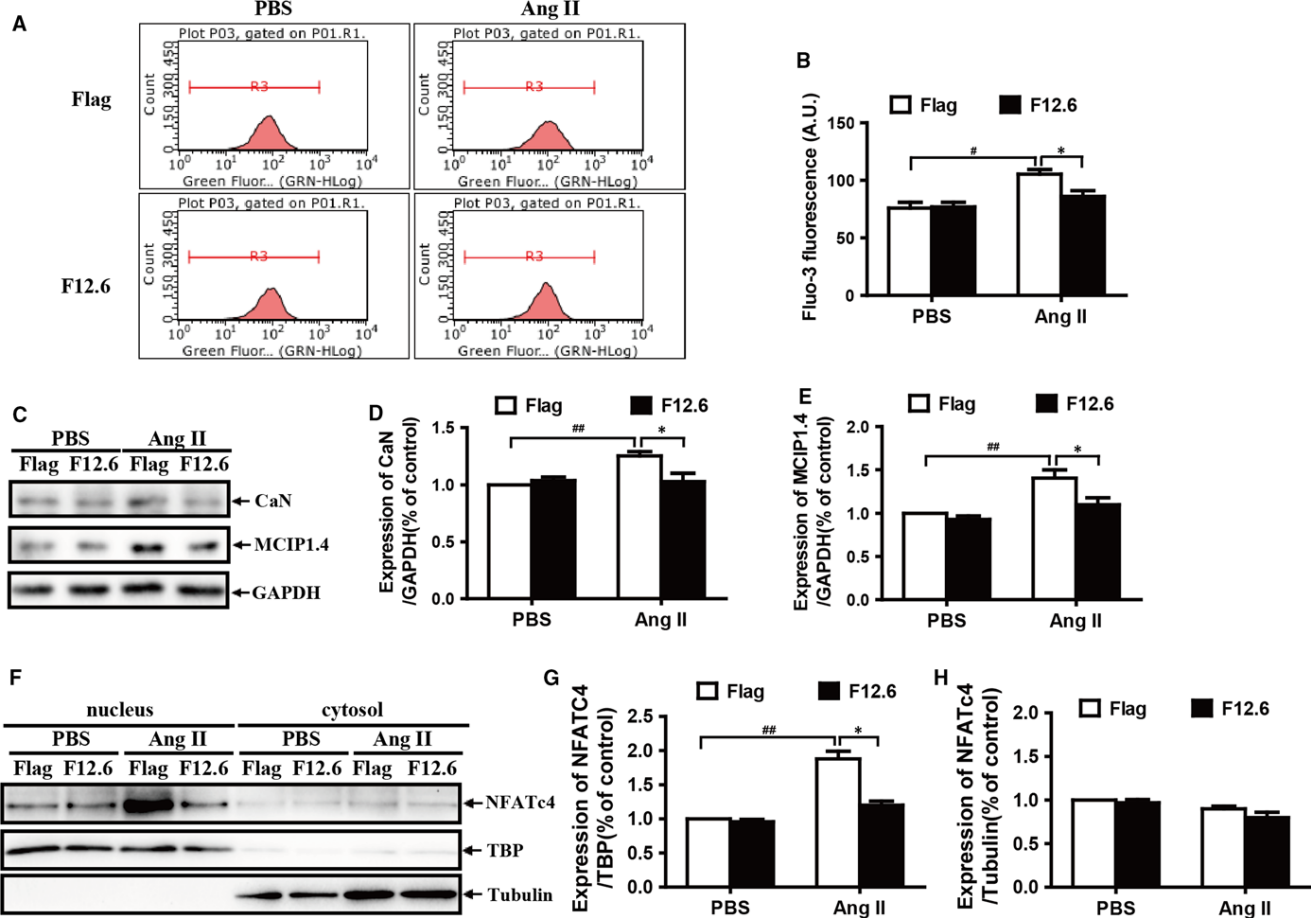
Overexpression of FKBP12.6 significantly reduces the elevation of the intracellular Ca^2+^ concentrations ([Ca^2+^]i) and inhibits the activities of Ca^2+^/calcineurin signalling pathways after AngII stimulation in H9c2 cells. The AngII‐induced elevation of the intracellular Ca^2+^ was detected by flow cytometric analysis in FKBP12.6 overexpression (F12.6) and Flag‐control (Flag) H9c2 cells with Fluo3‐AM staining after 20‐min PBS or AngII treatment (A) and quantitatively determined by measuring the mean fluorescence intensity of Fluo‐3 in each group (B). FKBP12.6 overexpressing (F12.6) and Flag‐control (Flag) H9c2 cells were treated with or without 200 nmol/L AngII for 24 h. The expressions of calcineurin (CaN) and MCIP1.4 proteins were detected by Western blot (C) and quantitatively determined by density analysis (D and E), respectively. The NFATc4 protein levels in cytosolic and nuclear fractions were analysed by Western blot (F). TATA‐binding protein (TBP), a nuclear protein marker, and Tubulin, a cytosolic protein marker, were used as an internal control, respectively. The contents of NFATc4 protein in nuclei (G) and cytosole (H) were quantitatively determined by density analysis. The data represent the mean ± SEM from three independent experiments, *P < .05 for FKBP12.6‐overexpression vs Flag‐control and ^#^ *P *< .05, ^##^ *P *< .01 for PBS vs AngII groups

As Ca^2+^ signalling plays a key role in AngII‐induced cardiac hypertrophy, whereas FKBP12.6 is involved in the regulation of Ca^2+^ release from SR, the concentrations of intracellular Ca^2+^ ([Ca^2+^]i) were detected with fluo3‐AM after AngII stimulation. The result showed that there was a markedly elevation of [Ca^2+^]i in Flag‐control H9c2 cells after AngII stimulation, in contrast, the [Ca^2+^]i in FKBP12.6 overexpression cells was much lower than that in Flag‐control cells (increased 11.8% vs 38.9%, *P < .05, n = 5; Figure 5A,B). These results suggested that the protection of FKBP12.6 overexpression on AngII‐induced hypertrophy might be associated with the reduction in the [Ca^2+^]i. Similar to the results in vivo, overexpressions of FKBP12.6 in H9c2 cells prevented the cells from the AngII‐induced the elevated expressions of calcineurin, MCIP 1.4 (Figure 5C‐E) and NFATc4 (Figure S3). Next, to further define the mechanism of calcineurin signalling pathway, the nuclear translocation of the cardiac form of NFAT (NFATc4) in the cells were determined. As showed in Figure 5F‐H, overexpression of FKBP12.6 in H9c2 cells markedly inhibited the AngII‐induced NFATc4 nuclear translocation (*P *< .05, n = 3).

### 3.6 Overexpression of FKBP12.6 significantly protects cardiomyocytes from the AngII‐induced hypertrophy through inhibiting the Ca^2+^/CaMKII, AKT/GSK3β and AKT/mTOR signalling pathways in H9c2 cells

**Figure 6:**
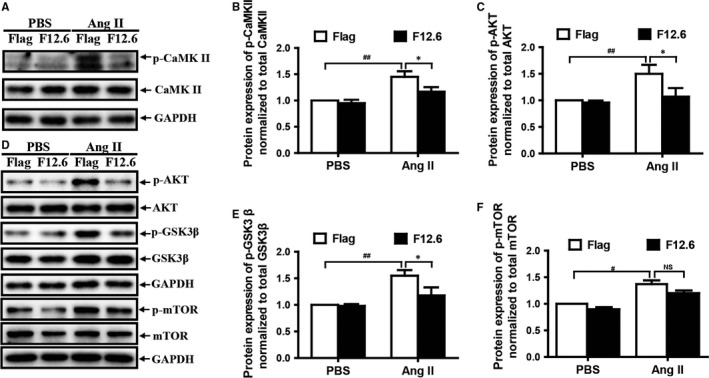
Overexpression of FKBP12.6 significantly protects cardiomyocytes from AngII‐induced hypertrophy through inhibiting the Ca^2+^/CaMKII, AKT/GSK3β and AKT/mTOR signalling pathways in H9c2 cells. FKBP12.6 overexpressing (F12.6) and Flag‐control (Flag) H9c2 cells were treated with PBS or with 200 nmol/L AngII for 20 min. The expressions of the total and phosphorylated CaMKII (A), AKT, GSK3β and mTOR (D) were detected by Western blot. The quantitative expressions of total and phosphoryated CaMKII, AKT and GSK3β protein were performed by density analysis, and the protein expression of p‐CaMKII (B), p‐AKT (C), p‐GSK3β (E) and p‐mTOR (F) was normalized to total CaMKII, AKT, GSK3β and mTOR. GAPDH was used as the loading controls. The data represent the mean ± SEM from three independent experiments, *P < .05, ***P < .001 for FKBP12.6‐overexpression vs Flag‐control and ^#^ *P *< .05, ^##^ *P *< .01 for PBS vs AngII groups

To further elucidate the mechanisms of the protection of FKBP12.6 on AngII‐induced cardiac hypertrophy, the effects of overexpressing FKBP12.6 on Ca^2+^/CaMKII, AKT/GSK3β and AKT/mTOR signalling pathways were examined. The results showed that, similar to the results in vivo, overexpressions of FKBP12.6 in H9c2 cells prevented the cells from the AngII‐induced the elevated expressions of the phosphorylated CaMKII (Figure 6A,B), phosphorylated AKT, phosphorylated GSK3β and phosphorylated mTOR, but there were no effects on the expressions of the total CaMKII, AKT, GSK3β and mTOR proteins (*P *< .05, n = 3) (Figure 6C‐F). These results demonstrated that overexpressing FKBP12.6 protects cardiomyocyte from the AngII‐induced hypertrophy through inhibiting the activities of Ca^2+^/CaMKII, AKT/GSK3β and AKT/mTOR pathways.

### 3.7 Overexpression of FKBP12.6 attenuates AngII‐induced myocardial apoptosis in H9c2 cells

**Figure 7:**
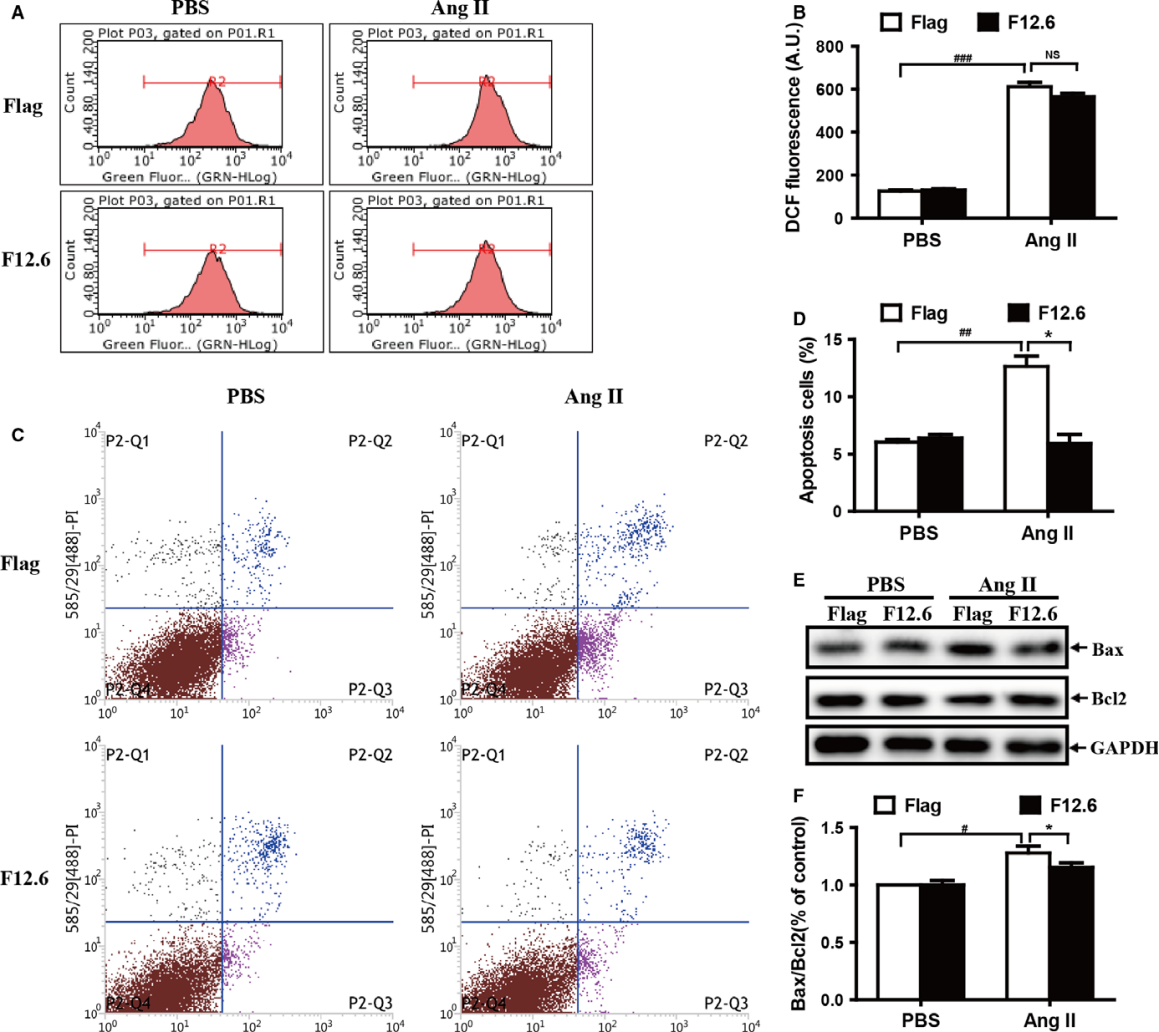
Overexpression of FKBP12.6 attenuates AngII‐induced myocardial apoptosis in H9c2 cells. FKBP12.6 overexpressing (F12.6) and Flag‐control (Flag) H9c2 cells were treated with PBS or 200 nmol/L AngII for 20 min. The intracellular ROS productions were measured by a flow cytometry stained with CM‐H2DCFDA (A) and the mean DCF‐fluorescence intensity in different group of cells was quantitatively determined (B). Cell apoptosis was detected by a flow cytometry with annexin V‐FITC/propidium iodide (PI) double staining. Representative flow cytometric dot plots (*x*‐axis: annexin V staining/*y*‐axis: PI staining) show two‐cell populations: annexin V/PI‐negative cells (normal cells, lower left quadrant) and annexin V‐positive/PI‐negative cells (early apoptotic cells, lower right quadrant) and annexin V‐positive/PI‐negative cells(late apoptotic cells, lower right quadrant) (C). The annexin V‐positive/PI‐negative cells (apoptosis cells) are shown in (D). The expressions of Bax and Bcl2 proteins were detected by Western blot (E) in FKBP12.6 overexpression (F12.6) and Flag‐control (Flag) H9c2 cells after treated with AngII (200 nmol/L) for 24 h, and the Bax/Bcl2 ratios were quantitatively determined by density analysis (F). GAPDH was used as an internal control. The data represent the mean ± SEM from three independent experiments, *P < .05 for FKBP12.6‐overexpression vs Flag‐control and ^#^ *P *< .05 for PBS vs AngII group, NS represents no significance

It has been reported that AngII promotes ROS generation and calcium overload. Severe oxidative stress could lead to the apoptosis of myocardial cells and subsequently activate the TGFβ‐Smad pro‐fibrosis signalling pathway.^28^, ^29^ As showed in Figure 7A,B, overexpression of FKBP12.6 had no significant effects on the ROS generation induced by AngII in FKBP12.6 overexpression H9c2 cells (*P *> .05, n = 3). However, overexpressing of FKBP12.6 protected cells from apoptosis induced by AngII (Figure 7C,D), promoted the expression of the antiapoptotic protein Bcl‐2 and inhibited the expression of the pro‐apoptotic protein Bax (Figure S7). Moreover, overexpression of FKBP12.6 significantly inhibited the ratio of Bax/Bcl‐2 after treatment with AngII in H9c2 cells (Figure 7E,F; *P *< .05, n = 3), indicating that FKBP12.6 may also protect cells from apoptosis induced by AngII stimulation. However, consistent to the in vivo studies, FKBP12.6 overexpression had a minor effect in the activation of TGF‐Smad3‐ERK1/2 fibrosis signalling pathway induced by Ang II administration (Figure S4) (*P *> .05, n = 3).

## 4 DISCUSSION

In vivo studies showed that the cardiac‐specific overexpression of FKBP12.6 protected heart from cardiac hypertrophy.^15^, ^16^, ^17^ In contrast, a few studies showed that the increased FKBP12.6 expression might be associated with some cardiovascular diseases, suggesting that the heart may employ FKBP12.6 up‐regulation as an adaptive response to ameliorate cardiac dysfunction.^30^ However, an in vitro study showed that the adenovirus‐mediated overexpression of FKBP12.6 induced hypertrophy and apoptosis in cultured neonatal cardiomyocytes due to the activation of ERK1/2,^18^ indicating that the role of FKBP12.6 in cardiac hypertrophy is still unclear. In the present study, using both overexpression and knockout mouse and overexpression cell models, we first time demonstrated that FKBP12.6 protects hearts from AngII‐induced hypertrophy in vivo and in vitro, seen as lack of FKBP12.6 gene markedly aggravate AngII‐induced cardiac hypertrophy, whereas cardiac‐specific overexpression of FKBP12.6 prevents the hypertrophic response to AngII. Moreover, we observed that there was no change in the cardiomyocyte sizes between the Flag‐control (Flag) and overexpression of FKBP12.6 (F12.6) H9c2 cells. We also found FKBP12.6 overexpression did not promote the activation of ERK1/2 as reported in adenovirus‐mediated overexpression of FKBP12.6,^18^ and it did not prevent the activation of ERK1/2 induced by AngII neither (Figure S4). Therefore, the different results may be due to using different sources of cardiomyocytes or different approaches to explore the role of the FKBP12.6 overexpression. It has been reported that H9c2 cells can accurately mimic the hypertrophic responses of primary rat neonatal cardiomyocytes in vitro following stimulation with hypertrophic factors such as angiotensin II or endothelin‐1, seen as an increase in cellular footprint combined with rearrangement of cytoskeleton and induction of foetal heart genes.^31^ H9c2 cells are rat heart embryonic myoblasts, with skeletal muscle properties, which terminally differentiate by fusing and forming multinucleated myotubes. Cardiomyocytes isolated from embryonic or neonatal rat hearts spread quite readily on the culture plate in 1% horse serum.^32^ We cultured H9c2 cells in 2% serum medium for a short time (no more than 48 hours) and at the end of our experiments, we did not observe that the cells differentiate into myocytes/myotubes. We showed that the protection of FKBP12.6 on the AngII‐induced cell hypertrophy was associated with inhibitions of the Ca^2+^‐mediated calcineurin/NFATc4, CaMKII/MEF‐2, and AKT/mTOR, AKT/GSK3β/NFATc4 signalling pathways.

Many pieces of evidences suggest that AngII elevates blood pressure, which in turn promotes haemodynamic overload. The adaptation of the heart to this overload is to alter cardiac protein expression and substantial cardiac remodeling.^2^, ^3^ In addition to vasoconstrictor effects, AngII also exerts direct growth‐promoting effects on cardiac tissues, resulting in myocardial hypertrophy and mechanical dysfunction independently on blood pressure elevation.^3^, ^33^, ^34^, ^35^, ^36^ Both physiological and pathological cardiac hypertrophies are associated with elevation of cardiomyocyte Ca^2+^ levels,^37^ whereas the alternations of intracellular Ca^2+^ modulate the activities of various Ca^2+^‐dependent signalling pathways including calcineurin/NFATc4 and CaMKII/MEF‐2 signalling pathways.^38^, ^39^

Activated calcineurin is both necessary and sufficient to induce cardiac hypertrophy in vitro and in vivo, cardiac‐specific overexpression of calcineurin in mice showed a dramatic increase in heart sizes. Increased activity of calcineurin results in dephosphorylation of NFATc4, and subsequently induces transcription of foetal cardiac genes *via* a combinatorial mechanism involving its direct interaction with GATA4.^40^ Our results showed that overexpression of FKBP12.6 significantly inhibited AngII‐induced calcineurin activities and NFATc4 nuclear translocation, suggesting that overexpression of FKBP12.6 prevents hearts or H9c2 cells from AngII‐induced hypertrophy by suppressing the activation of calcineurin/NFATc4 pathway. CaMKII induces phosphorylation of the class II histone deacetylase 4 (HDAC4), which in turn dissociates from the MEF‐2 transcription factor, and translocates to the cytoplasm from the nucleus, leading to the activation of MEF‐2, which is sufficient to promote pathological hypertrophy.^38^ In our study, overexpression of FKBP12.6 inhibited the AngII‐induced phosphorylation of CaMKII (indicative of CaMKII activation) in heart or in H9c2 cells, suggesting that overexpression of FKBP12.6 prevents hearts or H9c2 cells from AngII‐induced hypertrophy by suppressing the activation of CaMKII.

Recent studies showed that the mTOR signalling pathway plays a major role in modulating structural and functional adaptation of the heart to both haemodynamic stress and non‐haemodynamic factors.^41^ Rapamycin acts by irreversibly binding FKBP12 and FKBP12.6, which inhibits mTORC1 assembly, diminished hypertrophic responses to the Gq‐protein‐coupled receptor agonists AngII.^42^ AKT, a member of the AKT/PKG/PKC family of serine/threonine kinases, is known to function both upstream as an activator of mTOR and downstream as a phosphorylation target of mTOR at S473.^43^ Our results showed that overexpression of FKBP12.6 appears to be able to prevent AngII‐induced the increases of the phosphorylation‐AKT and phosphorylation‐mTOR and then inhibited the progress of cardiac hypertrophy induced by AngII.

GSK3β, as an inhibitor of hypertrophic signalling in the intact myocardium, is a ubiquitous serine‐threonine protein kinase which phosphorylates NFAT proteins and antagonizes the action of calcineurin by promoting NFAT nuclear export, and the activity of GSK3β is regulated by the phosphorylation status of serine‐9. AKT has been shown to phosphorylate serine‐9 of GSK3β, thereby inactivating the enzyme.^12^ As we expected, the phosphorylations of AKT and GSK3β were increased in response to stimulation of AngII in Flag‐control cells, indicating that the AngII‐induced decreased activity of GSK3β result in NFATc4 accumulated in the nuclear and promoted the development of cardiac hypertrophy, whereas FKBP12.6 overexpression significantly ameliorated such alterations. These results suggested that inhibition of FKBP12.6 overexpression on the AKT/GSK3β/NFATc4 signalling pathway further enhances the protection of the protein on AngII‐induced cardiomyocyte hypertrophy.

Evidences have showed that AngII‐induced oxidative stress preserve an important role in pathological hypertrophy. AngII activates membrane NAD(P)H oxidases, such as superoxide and hydrogen peroxide (H_2_O_2_) to produce ROS, which is involved in the pleiotropic effects of AngII.^3^ ROS generation may further induce cell apoptosis, which is involved in antiapoptotic Bcl2 and the pro‐apoptotic Bax.^44^ The apoptotic cells induced by AngII are then replaced by excessive collagen fibrosis, and reduced capillary density increased oxygen diffusion distances leading to myocardial ischaemia, and finally contribute to the transition from hypertrophy to failure.^28^ As showed in our study, overexpression of FKBP12.6 significantly reduced the apoptosis cells and inhibited intracellular Bax/Bcl‐2 ratio, but almost has no effects on AngII‐induced ROS generation, suggesting that overexpression of FKBP12.6 protects heart from hypertrophy partially through inhibiting apoptosis induced by AngII.

In addition, AngII‐induced oxidative stress and cell injury were reported to promote cardiac remodelling through TGFβ1 signalling pathway.^45^ AngII and TGFβ1 do not act independently from one another but rather act as part of a network that promotes cardiac remodeling.^46^ TGFβ, a potent stimulator of collagen production by cardiac fibroblasts, acts downstream of AngII and promotes myocyte growth and fibrosis.^46^ In the canonical pathway, Smad2/3 is phosphorylated by TGFβ receptor type 1. There is growing evidence in the literature to suggest that Smad2 is not a mediator of cardiac fibrosis, whereas Smad3 regulates of the secretory phenotype of myofibroblasts and has a detrimental effect on myocardial fibrosis.^46^, ^47^ We showed that FKBP12.6 overexpression did minor things in inhibiting the increased level of TGFβ1 expression, and phosphorylation‐Smad3 induced by AngII. Additionally, it is reported that the TGFβ/Smad pathway activates the transcription of several key fibrotic genes through the ERK1/2 pathway.^29^ ERK1/2 has been implicated as the regulators of cardiac hypertrophy in both cell culture and genetically modified mouse models. However, genetic inhibition of cardiac ERK1/2 promotes stress‐induced apoptosis and heart failure but has no effect on hypertrophy in vivo,^48^ and ERK1/2 pathway is reported to activate the transcription of several key fibrotic genes through TGFβ‐Smad pathway,^29^ indicating that ERK1/2 may not be necessary for cardiac hypertrophy but play an important role in promoting fibrosis. Our results showed that although FKBP12.6 overexpression did not promote the activation of ERK1/2 as reported in adenovirus‐mediated overexpression of FKBP12.6,^18^ it did not prevent the activation of ERK1/2 induced by AngII neither. Therefore, we speculated that FKBP12.6 overexpression is not sufficient to inhibit the ERK1/2‐TGFβ1‐Smad pathway for protecting hearts from AngII‐induced fibrosis.

## 5 CONCLUSION

Our results demonstrated that FKBP12.6 protects heart from AngII‐induced cardiac hypertrophy through preventing the activations of the Ca^2+^/calmodulin‐dependent signalling pathways such as calcineurin/NFATc4, CaMKII/MEF‐2, AKT/GSK3β/NFATc4 and AKT/mTOR pathways. Obviously, our findings strongly support that FKBP12.6 plays a protective role in the development of cardiac hypertrophy.

## CONFLICT OF INTEREST

The authors declare no competing financial interests.
