# Supplementary material for: FKBP12.6 protects heart from AngII‐induced hypertrophy through inhibiting Ca2+/calmodulin‐mediated signalling pathways in vivo and in vitro
Source: J Cell Mol Med. 2018 Apr 22;22(7):3638–51. doi: 10.1111/jcmm.13645 (PMC6010737; doi:10.1111/jcmm.13645)
Supplement: Supplementary file 1 [file JCMM-22-3638-s001.docx]

**Supplementary materials**

**FKBP12.6 protects heart from AngII-induced myocyte hypertrophy through inhibiting Ca^2+^/calmodulin-mediated signaling pathways *in vivo* and *in vitro***

Yun-Fei Xiao^1,2,*^, Zhi-Xiong Zeng^1,2,*^, Xiao-Hui Guan^1^, Ling-Fang Wang^1,2^, Chan-Juan Wang^1^, Huidong Shi^3^, Weinian Shou^4^, Ke-Yu Deng^1,#^, Hong-Bo Xin^1,2,#^

**Supplementary Table**1**.** Echocardiography FKBP12.6^-/-^ and WT male mice treated with AngII infused.

| Measurement | WT Saline | FKBP12.6^-/-^ Saline | WT AngII | FKBP12.6^-/-^ AngII |
| --- | --- | --- | --- | --- |
| IVS D, mm | 0.813±0.08 | 0.802±0.07 | 1.079±0.10^##^ | 1.224±0.08^$^ |
| IVS S, mm | 1.266±0.10 | 1.276±0.13 | 1.529±0.14^##^ | 1.760±0.16^$^ |
| LVID D, mm | 3.395±0.29 | 3.411±0.20 | 3.135±0.30 | 3.046±0.29 |
| LVID S, mm | 2.201±0.21 | 2.250±0.17 | 1.690±0.32 | 1.479±0.26 |
| LVPW D, mm | 0.834±0.09 | 0.884±0.05 | 1.102±0.14 | 1.147±0.14 |
| LVPW S, mm | 1.291±0.13 | 1.337±0.08 | 1.743±0.23 | 1.791±0.18 |
| EF,% | 65.68±7.00 | 64.13±4.45 | 78.80±6.93 | 83.93±5.00 |
| FS,% | 35.17±4.33 | 34.04±3.47 | 46.09±6.78 | 51.44±5.88 |
| LV mass, mg | 93.94±9.96 | 97.83±9.33 | 126.0±10.21^##^ | 137.9±13.96 |
| BW, g | 23.67±1.43 | 23.62±1.36 | 23.71±1.85 | 23.53±1.07 |
| HW, mg | 113.76±6.43 | 108.58±10.23 | 138.18±5.24 | 155.08±4.00 |
| HW/BW, mg/g | 4.70±0.28 | 4.91±0.33 | 5.98±0.34^##^ | 6.61±0.38^$^ |

IVS D, Interventricular septum diastolic thickness; IVS S, Interventricular septum systolic thickness; LVID D, left ventricular internal diastolic dimension; LVID S, left ventricular internal systolic dimension; LVPW D, left ventricular posterior wall diastolic thickness; LVPW S, left ventricular posterior wall systolic thickness; EF%, the ratio of ejection fraction; FS%, the ratio of fractional shortening; LV mass, left ventricular mass; BW, body weight; HW, heart weight; HW/BW, heart weight/ body weight. The data represent the mean ± SEM, n = 5, ^$^*P* < 0.05 for FKBP12.6^-/-^ *versus* WT and ^#^*P* < 0.05, ^##^*P* < 0.01 for saline *versus* AngII infusion groups.

**Supplementary Table 2.** Echocardiography in AngII infused FKBP12.6 TG and WT male mice

| Measurement | WT Saline | FKBP12.6 TG Saline | WT AngII | FKBP12.6 TG AngII |
| --- | --- | --- | --- | --- |
| IVS D, mm | 0.842±0.09 | 0.844±0.09 | 1.096±0.10^##^ | 0.960±0.08* |
| IVS S, mm | 1.306±0.11 | 1.301±0.10 | 1.587±0.11^##^ | 1.426±0.10* |
| LVID D, mm | 3.405±0.38 | 3.484±0.34 | 3.185±0.30 | 3.222±0.13 |
| LVID S, mm | 2.301±0.35 | 2.404±0.31 | 1.718±0.32 | 1.961±0.18 |
| LVPW D, mm | 0.814±0.18 | 0.823±0.17 | 1.125±0.24^#^ | 1.084±0.27 |
| LVPW S, mm | 1.191±0.13 | 1.209±0.12 | 1.768±0.28^##^ | 1.617±0.21 |
| EF,% | 61.89±7.03 | 59.76±6.38 | 78.71±6.93 | 70.94±7.40 |
| FS,% | 32.42±4.52 | 31.00±4.12 | 46.06±6.78 | 39.14±6.67 |
| LV mass, mg | 95.11±8.82 | 99.57±9.45 | 132.6±10.01^##^ | 118.8±8.68* |
| BW, g | 24.40±1.79 | 23.35±1.92 | 24.19±1.85 | 24.29±0.92 |
| HW, mg | 118.97±6.21 | 112.37±8.28 | 146.71±9.98 | 131.46±6.48 |
| HW/BW, mg/g | 4.887±0.29 | 4.825±0.26 | 6.080±0.34^##^ | 5.423±0.36* |

IVS D, Interventricular septum diastolic thickness; IVS S, Interventricular septum systolic thickness; LVID D, left ventricular internal diastolic dimension; LVID S, left ventricular internal systolic dimension; LVPW D, left ventricular posterior wall diastolic thickness; LVPW S, left ventricular posterior wall systolic thickness; EF%, the ratio of ejection fraction; FS%, the ratio of fractional shortening; LV mass, left ventricular mass; BW, body weight; HW, heart weight; HW/BW, heart weight/ body weight. The data represent the mean ± SEM, n = 5, ^*^*P* < 0.05 for FKBP12.6 TG *versus* WT and ^#^*P* < 0.05, ^##^*P* < 0.01 for saline *versus* AngII infusion groups.


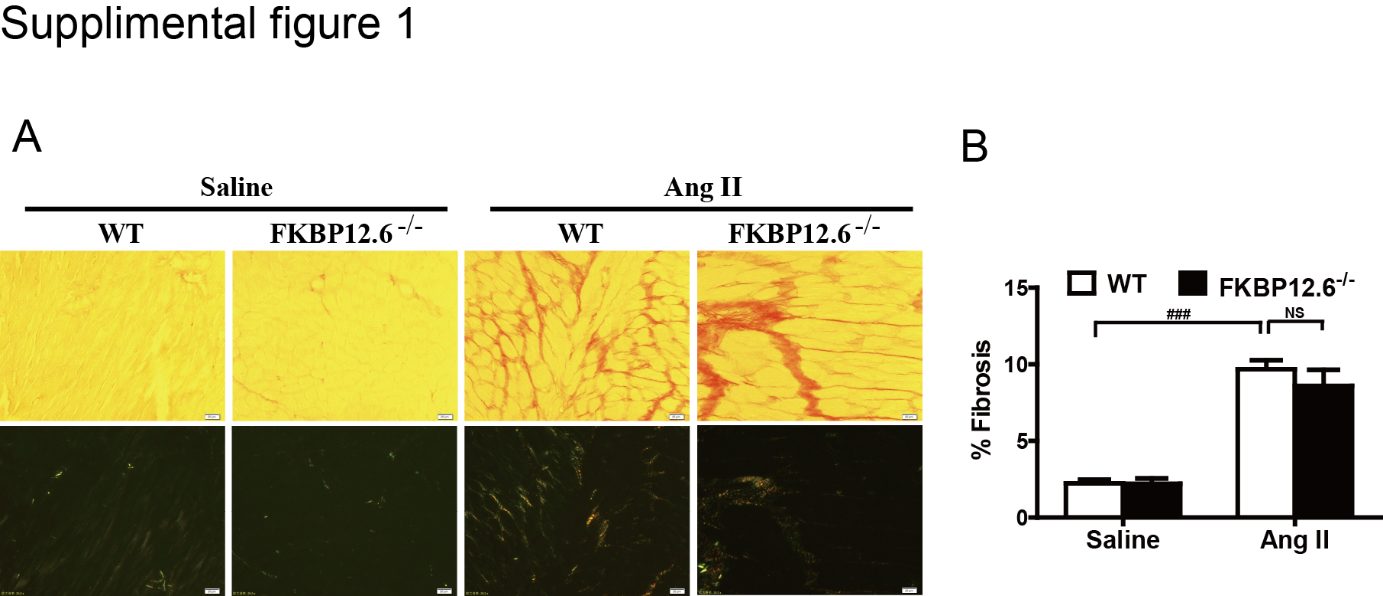


**Supplementary figure 1. Effects of FKBP12.6 deficiency on AngII-induced cardiac fibrosis.** FKBP12.6^-/-^ and WT mice were subjected to 14 days AngII or saline infusion. The collagen type I (orange) and III (green) contents **(A)** of WT and FKBP12.6^-/-^ hearts were stained by Picrosirius red under light microscopy (×400, upper, red) and polarized light (×400, down, orange and green), respectively. The percentage of fibrosis in cross section of heart was quantitatively determined **(B)**. The data represent the mean ± SEM, n = 5, ^###^*P* < 0.001 for saline *versus* AngII infusion groups, NS represents no significance.


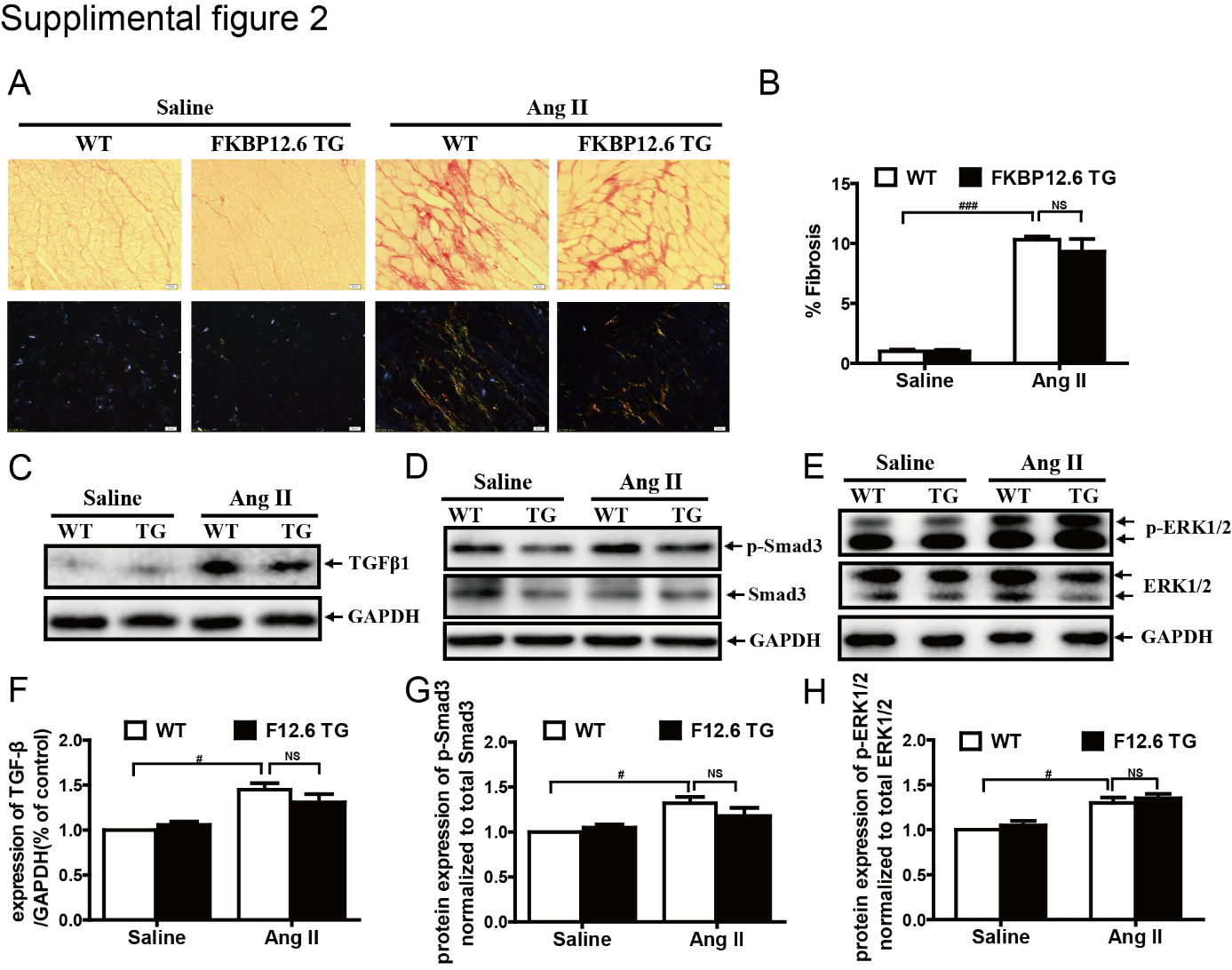


**Supplementary figure 2. Effects of Cardiac specific over-expression of FKBP12.6** **(FKBP12.6 TG) on AngII-induced cardiac fibrosis.** FKBP12.6 TG and WT mice were subjected to 14 days AngII or saline infusion. **(A)** The collagen type I (orange) and III (green) contents of WT and FKBP12.6 TG hearts were stained by Picrosirius red under light microscopy (×400, upper, red) and polarized light (×400, down, orange and green), respectively. **(B)** The percentage of fibrosis in cross section of heart was quantitatively determined. Western blot analysis of TGFβ1 **(C)**, p-Smad3 **(D)** and p-ERK1/2 **(E)** was performed in the hearts of FKBP12.6 TG and WT. quantification analysis of TGFβ1 (**F)** and was made. p-Smad3 **(G)** and p-ERK1/2 **(H)** was normalized to total Smad3 and ERK1/2. GAPDH was used as a loading control. The data represent the mean ± SEM, n = 5, ^#^*P* < 0.05 and ^###^*P* < 0.001 for saline *versus* AngII infusion groups, NS represents no significance.


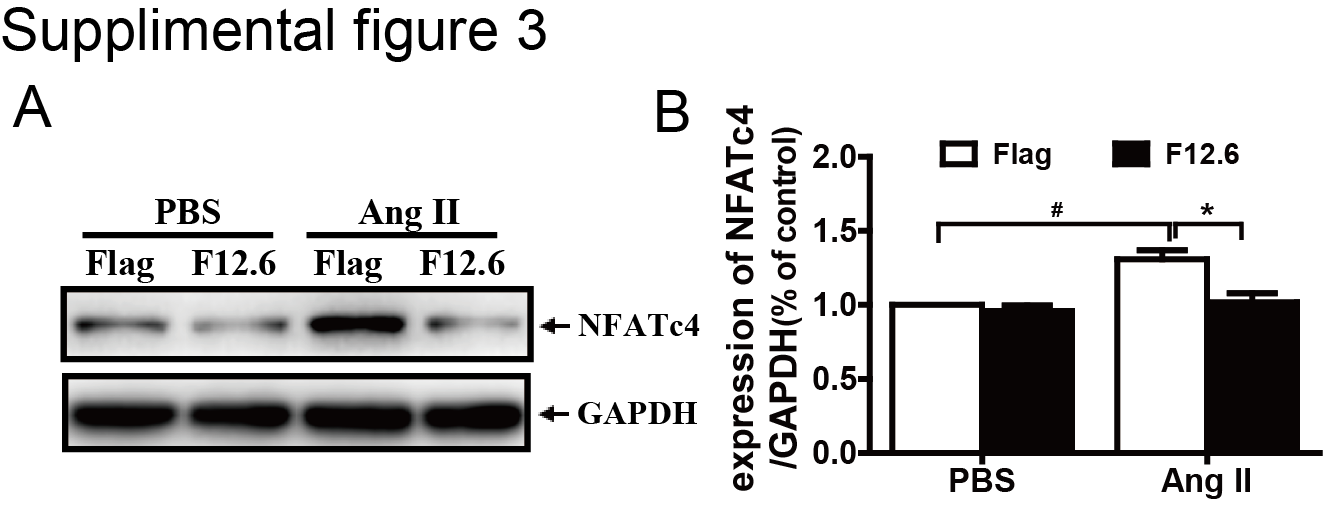


**Supplementary figure 3 (A)** Western blot analysis of NFATc4 performed in FKBP12.6 over-expression (F12.6) and Flag-control (Flag) H9c2 cells after treated with or without 200 nM AngII for 24 h. **(B)** Quantification analysis of NFATc4. GAPDH was used as a loading control. (Data are mean ± SEM for n = 3, ^#^*P* < 0.05 for PBS *versus* AngII stimulation groups, **P* < 0.05 for FKBP12.6 overexpression *versus* Flag-control groups.)


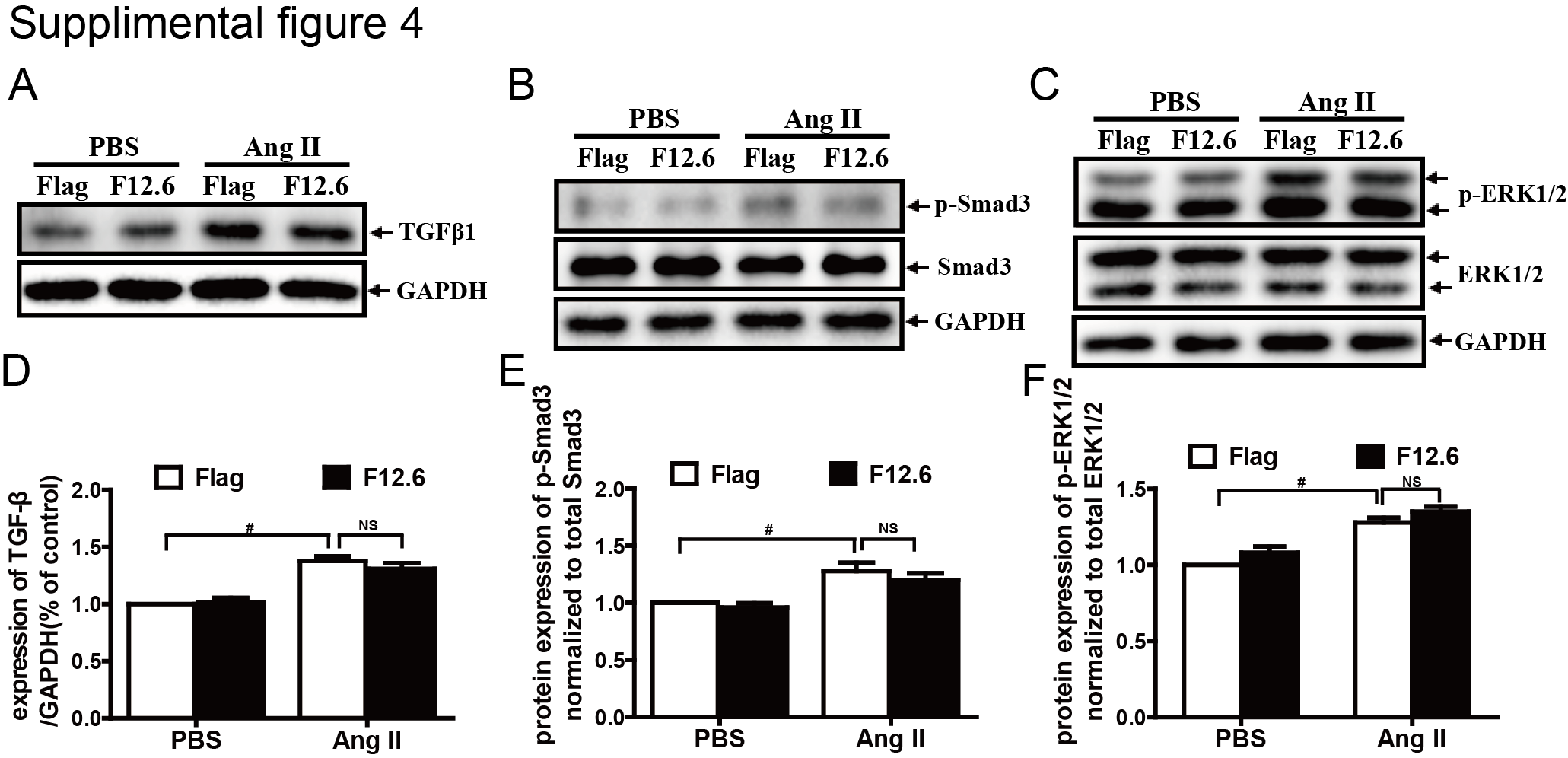


**Supplementary figure 4 (A)** Western blot analysis of TGFβ1 was performed in FKBP12.6 overexpression (F12.6) and Flag-control (Flag) H9c2 cells treated with or without 200 nM AngII for 24 h. Western blot analysis of p-Smad3**(B)** and p-ERK1/2**(C)** performed in FKBP12.6 overexpression (F12.6) and Flag-control (Flag) H9c2 cells after treated with or without 200 nM AngII for 20 min. quantification analysis of TGFβ1 **(D),** p-Smad3 **(E)** and p-ERK1/2 **(F)** was made. p-Smad3 and p-ERK1/2 was normalized to total Smad3 and ERK1/2. GAPDH was used as a loading control. (Data are mean ± SEM for n = 3, ^#^*P* < 0.05 for PBS *versus* AngII stimulation groups, NS represents no significance)


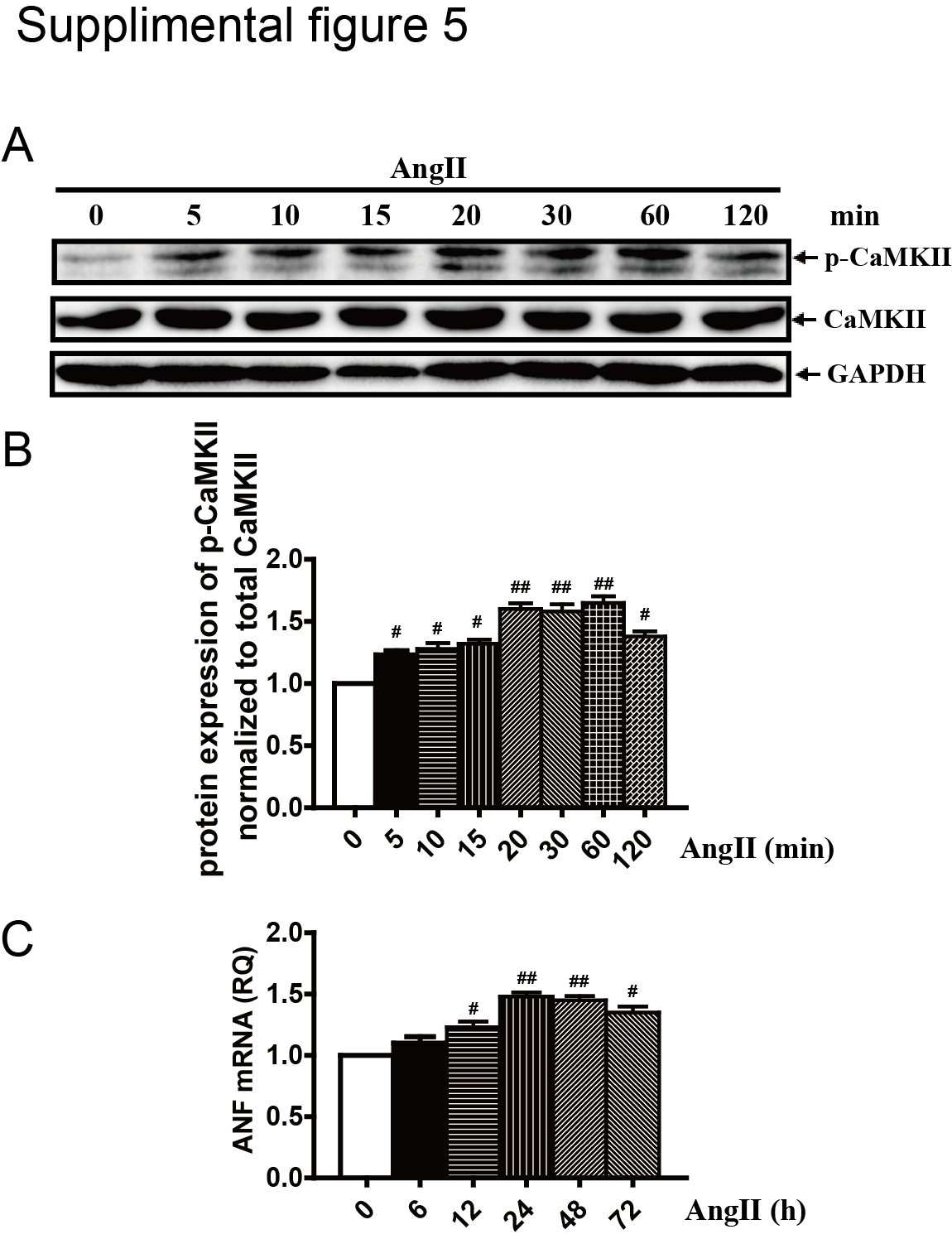


**Supplementary figure 5 (A)** Western blot analysis of p-CaMKII was performed in Flag-control (Flag) H9c2 cells after treated with 200 nM AngII for 0, 5, 10, 15, 20, 30, 60, 120 min. (B) expression of p- CaMKII was normalized to total CaMKII. GAPDH was used as a loading control. (C) mRNA analysis of ANF was performed by qRT-PCR in Flag-control (Flag) H9c2 cells after treated with 200 nM AngII for 0, 6, 12, 24, 48, 72 h. (Data are mean ± SEM for n = 3, # *P* < 0.05 and ## *P* < 0.01 for different time exposure to AngII *versus* 0 min or 0 h.)


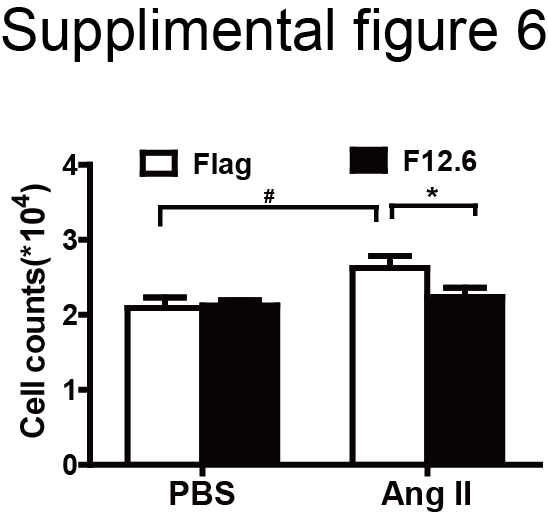


**Supplementary figure 6** Cell viability was determined by CCK8 (DOJINDO) according to the manufacturer’s instruction. Cells were seeded at 1 × 10^3^ cells/well in a 96 well plate, cell counts are determined by standard curve method after 24 h AngII treatment. (Data are mean ± SEM for n = 3, ^#^*P* < 0.05 for PBS *versus* AngII stimulation groups, **P* < 0.05 for FKBP12.6 overexpression *versus* Flag-control groups.)


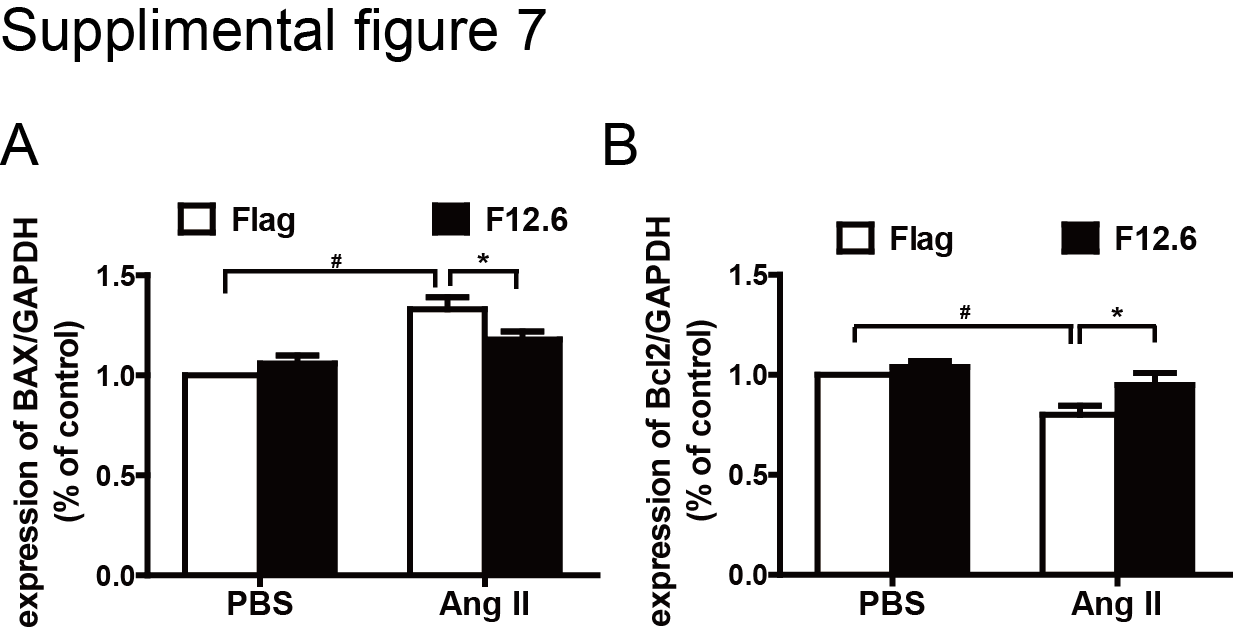


**Supplementary figure 7** FKBP12.6 overexpression (F12.6) and NC (Flag) H9c2 cells were treated with or without 200 nM AngII for 24 h. Quantification analysis of Bax **(A)** and Bcl2 **(B)**. GAPDH was used as a loading control. (Data are mean ± SEM for n = 3, ^#^*P* < 0.05 for PBS *versus* AngII stimulation groups, **P* < 0.05 for FKBP12.6 overexpression *versus* Flag-control groups.)
